# Maternal diabetes disrupts early corticogenesis through altered mitotic gene regulation: a transcriptomic analysis

**DOI:** 10.3389/fendo.2025.1564441

**Published:** 2025-05-13

**Authors:** Rocío Valle-Bautista, Diana S. de la Merced-García, Dafne A. Díaz-Piña, Néstor Fabián Díaz, Daniela Ávila-González, Anayansi Molina-Hernández

**Affiliations:** Departamento de Fisiología y Desarrollo Celular, Instituto Nacional de Perinatología ”Isidro Espinosa de los Reyes“, Mexico City, Mexico

**Keywords:** maternal diabetes, embryo cortical transcriptome, corticogenesis, neural stem cells mitosis, achromatic spindle

## Abstract

Maternal diabetes is linked to neurodevelopmental impairments in offspring, but the underlying molecular mechanisms remain unclear. Early cortical neurogenesis is a critical window vulnerable to maternal metabolic disturbances. Here, we analyzed global gene expression by RNA sequencing in dorsal prosencephalon tissue from 12-day-old embryos without neural tube defects. Gene ontology (GO) enrichment identified key candidates, validated by qRT-PCR, Western blotting, and immunofluorescence. We found 247 differentially expressed genes (111 upregulated, 136 downregulated), with upregulated genes enriched in mitosis, microtubule organization, and chromosome segregation pathways. Aurkb and Numa1 emerged as central regulators and were confirmed upregulated by qRT-PCR. Although Western blotting showed no protein-level changes, immunofluorescence revealed altered subcellular localization, disrupted spindle architecture, monopolar spindles, and increased asymmetric divisions in neural stem cells. These results suggest maternal diabetes disrupts mitotic regulation, accelerates neurogenic differentiation, and depletes the neural stem cell pool, potentially contributing to cortical defects and neurodevelopmental impairments in offspring. This study provides new insight into the developmental origins of neurodevelopmental disorders in the context of maternal diabetes, highlighting mitotic dysregulation as a potential mechanistic link in fetal programming.

## 1 Introduction

Maternal diabetes is associated with cognitive impairments in offspring, including verbal and nonverbal IQ scores, working memory and cognitive flexibility, and attention deficit hyperactivity disorder (ADHD) (1–5). The prefrontal cortex is essential in mediating these cognitive and executive functions, as it integrates information from various brain regions to support complex cognitive processes (6).

During corticogenesis, several alterations have been reported in embryos of diabetic rodents, including precocious and enhanced neurogenesis, reduced proliferation, a thinner cerebral cortex with impaired cytoarchitecture, and decreased excitability of deep-layer cortical neurons in 21-day-old pups (7–10). Furthermore, 14-day-old embryos (E14) from diabetic rats exhibited increased nuclear localization of FOXP2, a transcription factor implicated in speech and language development. During cortical development, FOXP2 promotes the transition from NSC population to intermediate progenitor (IP) and, ultimately, neuronal differentiation (7, 11). Among the targets of FOXP2 are genes involved in neurogenesis, cell death, and cell migration (12). The above suggests an accelerated NSC-to-IP transition.

Cortigogenesis is precisely regulated by intrinsic and extrinsic factors (13–15). Initially, multipotent epithelial stem cells proliferate to form a semi-stratified neuroepithelium, generating the first neurons, Cajal-Retzius cells, which delineate the basal side of the developing cortex. Within the semi-stratified neuroepithelium, radial glial cells (RGCs), a type of bipolar NSCs, undergo symmetrical and asymmetrical division to expand its pool or to produce neurons, astrocytes, and oligodendrocytes in a temporally controlled manner to determine the size and function of the brain (16). Neurons are the earliest specialized cells to arise, originating directly from NSCs or indirectly via IPs, and to establish the characteristic laminar structure of the cerebral cortex, they migrate in an inside-out pattern manner, with the deeper layer neurons born first and the more superficial layers at the end (13, 17–19). Typical in rodents, IPs undergo a terminal division, yielding two neurons. However, IP cells that undergo two successive divisions in the dorsal cortex are found at low frequencies (20).

During the NSC cell cycle, the nucleus undergoes interkinetic nuclear migration, moving between the apical and basal sides of the ventricular zone (VZ), moving basally during G1, undergoing the S-phase on the basal side, migrating apically during G2, and finally, on the apical surface, mitosis occurs (21).

Axis polarity, spindle orientation, and cleavage plane orientation are critical factors influencing whether divisions are symmetric or asymmetric. When the mitotic spindle aligns parallel to the cell’s apical-basal axis, asymmetric divisions are favored. Conversely, when it is perpendicular, division tends to be symmetric. Another factor regulating the transition from neuroepithelial cells to RGCs and from proliferative to neurogenic divisions is the length of the NSCs cycle, specifically an elongation of the G1 phase (22, 23). Intrinsic differences in mitotic spindle architecture at different neurogenic stages have also been documented. While in the early stages, an astral spindle morphology, with numerous and longer microtubules contacting the cellular cortex, in later stages, it loses its astral morphology and exhibits an increased density of inner spindle microtubules (24).

Although previous studies have provided insight into how maternal diabetes affects embryonic nervous system development, many investigations have not distinguished between embryos with and without neural tube defects. This lack of distinction makes it challenging to identify the underlying mechanism involved in postnatal neurodevelopment impairments in “normally” developing embryos. Nonetheless, valuable information has emerged, indicating changes in DNA methylation and the expression of genes associated with synaptic plasticity, neurotransmitter signaling, mitochondrial metabolism, neuroinflammation, neuronal development, synaptic function, cell proliferation, cytoskeletal remodeling, and oxidative phosphorylation (8, 25–28). To address part of these gaps, we separate embryos without neural tube defects and examine how high glucose influences the neural tube before the onset of the neurogenic peak, thereby focusing on the early cellular events that may affect cell fate determination.

Here, we analyzed the transcriptome from pooled total RNA samples obtained at E12 from the dorsal prosencephalon of control (Ctl) and diabetic (Db) pregnant rats. Our results showed that in the Db group, 247 genes were differentially expressed (111 up-regulated and 136 down-regulated) compared to the Ctl. Gene Ontology (GO) analysis of biological processes and cellular components revealed significant enrichment in pathways related to mitosis, microtubule organization, catalytic activity, and nuclear lumen. Notably, the genes Numa and Aurkb were listed in more than half of the enriched categories. Given their pivotal roles in the cell cycle and mitosis (29, 30), we validated their expression and evaluated protein levels. While qRT-PCR confirmed up-regulation of Numa and Aurkb in the Db group, no changes in total protein content were detected. However, immunofluorescence revealed altered distribution patterns of NUMA, phosphorylated AURKB (AURKBph), and α-tubulin (α-TUB) in the VZ. Furthermore, alterations in mitotic spindle architecture and an increase in asymmetric cell division in the Db group were also observed, underscoring the impact of maternal diabetes on early corticogenesis.

The findings presented here might advance our understanding of the molecular basis of neurological disorders associated with maternal diabetes. Article types

## 2 Materials and methods

All animal experiments and procedures followed ARRIVE guidelines 2.0 (31) and Mexican NOM-062-ZOO-1999. The protocol was approved by the Instituto Nacional de Perinatología committees of Research and Animal Care (CICUAL), Biosecurity, and Ethics (protocol number 2018-1-146).

### 2.1 Diabetes induction and embryonic tissue recovery

Adult female Wistar rats (250–310 g) were housed under standard conditions (12:12 h light/dark cycle, 21 ± 2°C and 40% relative humidity) with ad libitum access to food and water. Females were mated with fertile male rats during the dark cycle, and the presence of spermatozoids in a vaginal smear within the first four hours of the light cycle was considered E0.5. Pregnant rats were then housed in groups of five.

Pregnant rats were housed in groups of five and randomly assigned to Ctl or Db groups. At E5, the Ctl group received a single intraperitoneal injection of citrate buffer (pH 6.4, 250 μL), while the Db received a single injection of streptozotocin (STZ; 50 mg/kg in 250 μl vehicle; Sigma Aldrich, MO, USA). After 48 h, plasma glucose was measured from tail vein blood using an electronic glucometer (ACCU-Check Performa, Roche Diagnostics, Basel, CH). Only rats injected with STZ with blood glucose levels higher than 200 mg/dL at euthanasia were included in the Db group, and those that received the vehicle with glucose levels between 96-120 mg/dL were included in the Ctl group. Db pregnant rats in the study exhibited blood glucose levels of 428.3 ± 14.9 mg/dL, whereas control animals showed levels of 109 ± 10.2 mg/dL.

**Figure 1:**
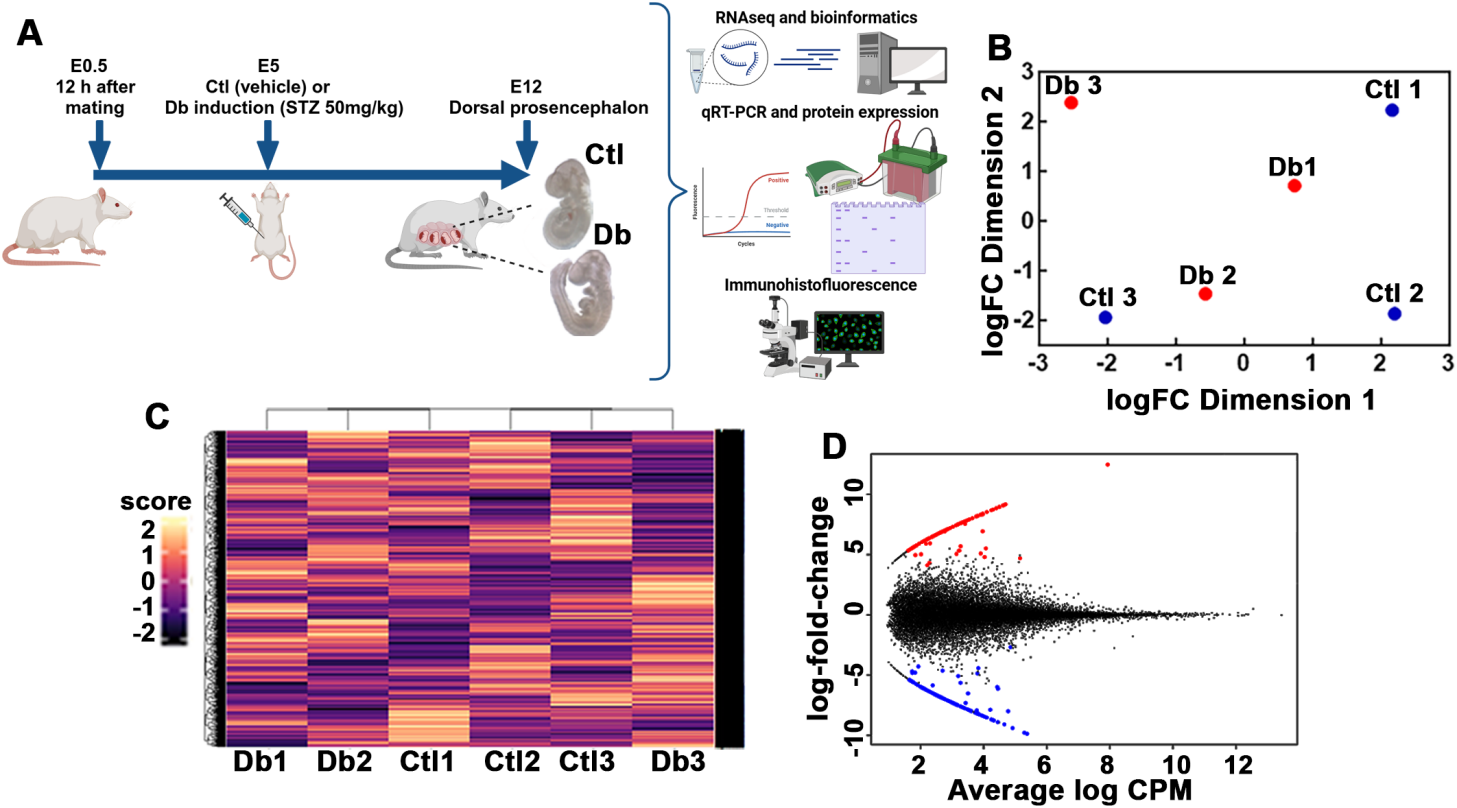
Experimental strategy and transcriptomic analysis of control (Ctl) and diabetic (Db) groups. **(A)** Schematic representation of the experimental strategy for analyzing transcriptomic differences between 12-day-old embryos (E12) from control (Ctl) and diabetic (Db) rats. Only embryos without neural tube defects were included. **(B)** Principal Component Analysis (PCA) plot showing distinct clustering of transcriptome data from Ctl (blue dots) and Db (red dots) groups of E12 dorsal prosencephalon tissue. **(C)** Hierarchical clustering heatmap of the transcriptomic data from Ctl and Db groups. Rows represent individual genes, and columns represent samples. Color intensity corresponds to expression levels, with distinct clustering reflecting differential gene expression between groups. **(D)** MA/SMEAR plot showing differential gene expression análisis between E12 tissue from Ctl and Db groups. Up-regulated (red dots) and down-regulated genes (blue dots) are highlighted (p-value < 0.05, and log2 fold change > 1.5).

At E12, pregnant rats were euthanized by decapitation following anesthesia induction with sevoflurane; embryos were collected by cesarian and washed in cold phosphate-buffered saline (PBS, pH 7.4). Only embryos without neural tube defects were included in further analyses (**Figure 1A**). Embryos with neural tube defects were identified under a stereomicroscope and excluded if they exhibited anencephalia, brain vesicle abnormalities (malformed or asymmetrical), aberrant neural tube closure in the forebrain or spinal cord, or abnormal and brain embryos with brains showing folding.

Embryos of both sexes were used without distinction in all experiments. As previously reported, the dorsal telencephalon of E14 embryos from diabetic pregnant rats exhibits increased neurogenesis, presumably due to an early shift in NSC differentiation either directly or via IP generation (7, 9). We selected E12 as it marks the onset of the cortical laminar neurogenic phase in rats, characterized by the initiation of asymmetric NSC division alongside ongoing active symmetric division. This developmental window is critical for establishing the initial cortical architecture and the neural progenitor pool, making it a relevant time point to evaluate the effects of maternal diabetes on early neurodevelopmental processes.

For RNA-seq (n = 3), qRT-PCR (n = 4), and Western Blot (WB), 30 dorsal prosencephalons per experimental unit for a total of an n = 3 were dissected under a stereoscopic microscope (Olympus Corporation S7X2-ILLT, Tokyo, JPN) and frozen at -70°C until used. For the immunofluorescence analyses, three embryos per litter per experimental unit (n = 3) were fixed in 4% paraformaldehyde (in PBS, pH 7.4) and processed for histology. For cell culture experiments, the dorsal prosencephalon from non-neural tube defective embryos of an entire litter or two litters, for control and diabetic groups, respectively (n = 1), were mechanically dissociated, and a total of 3 experiments were performed in passage one, as described below. The total number of animals used was 83 female and six male rats for mating.

### 2.2 RNA-seq and bioinformatic analysis

Total RNA was obtained using TRIZOL^®^ reagent (Thermo Fisher Scientific, MA, USA) following provider’s instructions. RNA quality and purity were determined (Nanodrop, Thermo Fisher Scientific), and only 260/230 ≥ 1.0 and 260/280 ≥ 1.8 samples were used. RNA Integrity Number (RIN) was obtained by using a BioAnalyzer (Agilent 2100 BioAnalyzer, CA, USA). The mean RIN was 9.04 ± 0.44.

RNA-seq libraries were prepared using the Truseq Stranded mRNA library prep kit from (Illumina, CA, USA) according to the manufacturer’s instructions. Sequencing was conducted on the Illumina Nextseq 500 platform with paired-end two lectures ×75 bp reads, cycles paired-end to generate approximately 30 million reads per sample.

Quality control of raw reads was performed with FastQC (32), and adapters were removed using Cutadapt (33). Trimmed reads were aligned to the Rattus_norvegicus reference genome (mRATBN7.2) using the Bowtie2 (34). Gene-level quantification was performed using RSEM v1.3.3 (35). Total counts for each sample were merged; a matrix was generated using the “abundance_estimates_to_matrix.pl” included in the Trinity pipeline (34).

Differential expression analysis was performed with edgeR (36). The abundance of all genes was calculated using the mapped reads by the Fragments per Kilobase of transcript per Million fragments mapped (FPKM) method, combined with RSEM to measure and normalize gene expression levels and to identify genes with significant differences in expression in the Db versus (vs) the Ctl group. For the up- and down-regulated genes, an ontology (GO) enrichment analysis was performed using the ShinyGO (v0.741) (37).

### 2.3 qRT-PCR analysis

cDNA was synthesized from one microgram of total RNA (Promega, WI, USA). PCRs were performed using a 10 µL mix containing cDNA (500 ng), forward and reverse primers (0.8 pmol), and NZYSpeedy qPCR Green Master Mix 2× (NZYTech, Lisbon, PRT. Primer sequences were: Aurkb, forward 5’-GTAGGTTCTCCGGTGTACGA-3’ and reverse 5’-AGGTGTTCAGGCCAGATTGA-3’; Numa, forward 5’-GGGGGATATGGAACGATGGG-3’ and reverse 5’-TAGTTAGAGACAGGGGCCAGA; and actin, forward 5’-CCGCGAGTACAACCTTCTTGC-3’ and reverse 5’-GTACTTCAGGGTCAGGATGCC-3’.

The PCRs conditions were: 10 min denaturalization at 95°C, 35 cycles of denaturalization (30 s at 95 °C), aligning (15 s; Aurkb 60°C, Numa 56°C, and actin 63°C), and extension (30 s at 72 °C). Actin was used as internal control, whereas total RNA and the PCR mix without cDNA were negative controls.

End-point PCR products were recovered using the ZymocleanTM Gel DNA Recovery Kit (Zymo Research, Irvine, CA, USA) and sequenced. Using BLAST^®^ (standard nucleotide BLAST), a 100% identity was obtained for each product (nucleotides 25-254 of NM_053749.2, 7036-7257 of NM_031144.3, and 18-285 of NM_031144.3 for Aurkb, Numa, and actin, respectively).

Efficiency and threshold values for each qPCR product were obtained from dynamic ranges using a Rotor-Gene thermocycler (QIAGEN, Venlo, NLD). Melting curves were performed to ensure a single amplified product. The 2^-ΔΔCT^ relative expression method was used to evaluate changes in gene expression between groups (24).

### 2.4 Western blot analysis

The tissue was homogenated in lysis buffer (20 mM HEPES, 1.5 mM MgCl2, 10 mM KCl, 1 mM DTT, and protease/phosphatase (Thermo Fisher Scientific; A32961). After centrifugation (10 min, 13000× rpm), the supernatant was collected, and protein concentration was determined by the Bradford method (38). The protein (60 µg) was separated in 10% SDS-PAGE gels using the MiniProtean II system (Bio-Rad, Hercules, CA, USA) and transferred to nitrocellulose membranes (AmershamTM Hybond TM-ECL, Buckinghamshire, UK) using a semi-dry transfer cell system (Bio-Rad) (39).

Membranes were blocked with TBS blocking buffer (LI-COR, Lincoln, NE, USA), incubated overnight at 4°C with rabbit anti-NUMA (1:500, GTX64368, GeneTex, Irvine, CA, USA), rabbit anti-AURK (1:1000, ab287960, Abcam, Cambridge, UK), and rabbit anti-AURKT232ph (1:1000, ab115793, Abcam), and mouse anti-actin as internal control (1:2000; GTX82559) antibodies, and incubated with IRDye 800CW donkey anti-rabbit and 680RD donkey anti-mouse (1:1000, P/N: 926-32213 and 926-68073, LI-CORbio, Lincoln, NE, United States) secondary antibodies. Bands were visualized using an Odyssey infrared scanner and analyzed using Image Studio ver.4.0 (LI-CORbio). The fluorescence rate of each protein was obtained, along with that of Actin.

### 2.5 Neural stem cell culture

Embryo tissue was mechanically dissociated in 1:1 N2 (DMEM/F12 (ATCC, Manassas, VA, USA), GlutaMAX, N2 supplement, 0.1 mM non-essential amino acids, 0.1 mM 2-mercaptoethanol, and 50 U/ml penicillin/streptomycin) and Neurobasal/B27 media (B27 supplement, two mM glutamine, and 50 U/ml penicillin-streptomycin), seeded (120,000 cells/well; 24-well plates) onto plates coated with 15 µg/ml poly-L-ornithine (Sigma-Aldrich) and 1 µg/ml fibronectin (Thermo Fisher), maintained at 37°C, 5% C02 and FGFb (R&D, Minneapolis, MN, USA), and the medium changed every third day until 80% confluency. Cells were passaged (150,000 cells/well) and, after 24 hours, fixed with 4% paraformaldehyde.

### 2.6 Immunofluorescence

Paraformaldehyde fixed consecutive coronal sections (10 μm thick) placed on poly-L-lysine coated slides from paraffin-embedded embryonic brains containing the dorsal prosencephalon obtained in a microtome (HM320, Thermo Fisher/Micron), dorsal prosencephalon explants, and cultured NSCs were used for immunofluorescence. Samples were permeabilized and blocked in PBS containing 0.3% Triton-X100 and 10% normal goat serum or 5% bovine serum albumin for 30 min, incubated overnight at 4°C with primary antibodies (rabbit anti-NUMA, 1:50; rabbit anti-AURKB, 1:250; rabbit anti-α-AURKT232ph, 1:250; and mouse anti-α-Tubulin, ab7291, 1:250), one hour with Alexa Fluor IgG 488 anti-rabbit and 568 anti-mouse (1:1000; A-11008 and A-11004, Thermo Fisher Scientific) secondary antibodies, nuclei were stained with DAPI (one ng/mL; Sigma-Aldrich), and mounted in AquaPolymount. Controls omitting the primary antibody were included. Explants were placed in an en-face view (40). Confocal images were obtained using a Leica TCS-SP8 DM6000 (Leica, Leitz, DEU) microscopy with a 40× (N.A. 1.3) objective for slices and a Leica TCS-SPE DMI4000 (Leica) microscope with a 63× (N.A.1.2) objective for explants. Representative images are shown as single confocal z-stack images. NSC images were obtained using an Olympus IX81 epifluorescence microscope and a Hamamatsu ORCA-Flash 2.8 CCD camera (Hamamatsu, JPN), deconvoluted using ImageJ. Representative images were processed using Adobe Photoshop CS6 (Adobe Inc., San Jose, CA, USA).

### 2.7 Achromatic spindle measurement

The length of achromatic spindles in cultured E12 NSC was measured using FIJI software (ImageJ) with the Analyze Skeleton plugin (https://imagej.net/plugins/analyze-skeleton/). Micrographs (40×) were converted to binary, scale set in µm, and the following steps were performed: Analyze>Skeleton>Classify particles using skeleton. AURKBT232ph mark was selected for the particle image and the α-TUB for the skeletonizable mask parameters. MaxEntropy was used to detect auto-threshold particles (41). The resulting skeleton structure for each achromatic spindle was used to obtain the total size and the “Longest-Shortest Path”. As the mitotic spindles showed a complex skeleton with numerous “branches”, the tagged skeleton was represented as an undirected and weighted graph, where the nodes are the end-point and junction pixels, and the weighted edges are the summed Euclidean distances between every slab pixel and its neighbor in the edge (42). For our purpose, the longest-shortest path was used to obtain the size of the mitotic spindle.

### 2.8 Statistics analysis and graphs

Differentially expressed genes were identified with a false discovery rate (FDR) < 0.05 and at least a 1.5-fold change in FPKM values between groups. Data are mean ± standard error of the mean (SEM). Differences between groups were assessed by unpaired Student’s *t*-test (*P<*0.05 considered significant). Statistics and graphs were performed in GraphPad Prism version 9 (GraphPad Software, Inc., San Diego, CA, USA).

## 3 Results

### 3.1 Bioinformatic analysis

All samples showed FastQC scores above 28. Principal component analysis (PCA) indicated that two samples from each group clustered closely together, highlighting the variability in gene expression among E12 embryos (**Figure 1B**). Hierarchical clustering analysis further supported these findings, showing that two diabetics and two control samples grouped together while one sample from each group diverged from the main cluster (**Figure 1C**). Despite the heterogeneity, all samples passed quality control, and thus, all were included in the differential gene expression and subsequent analysis.

**Figure 2:**
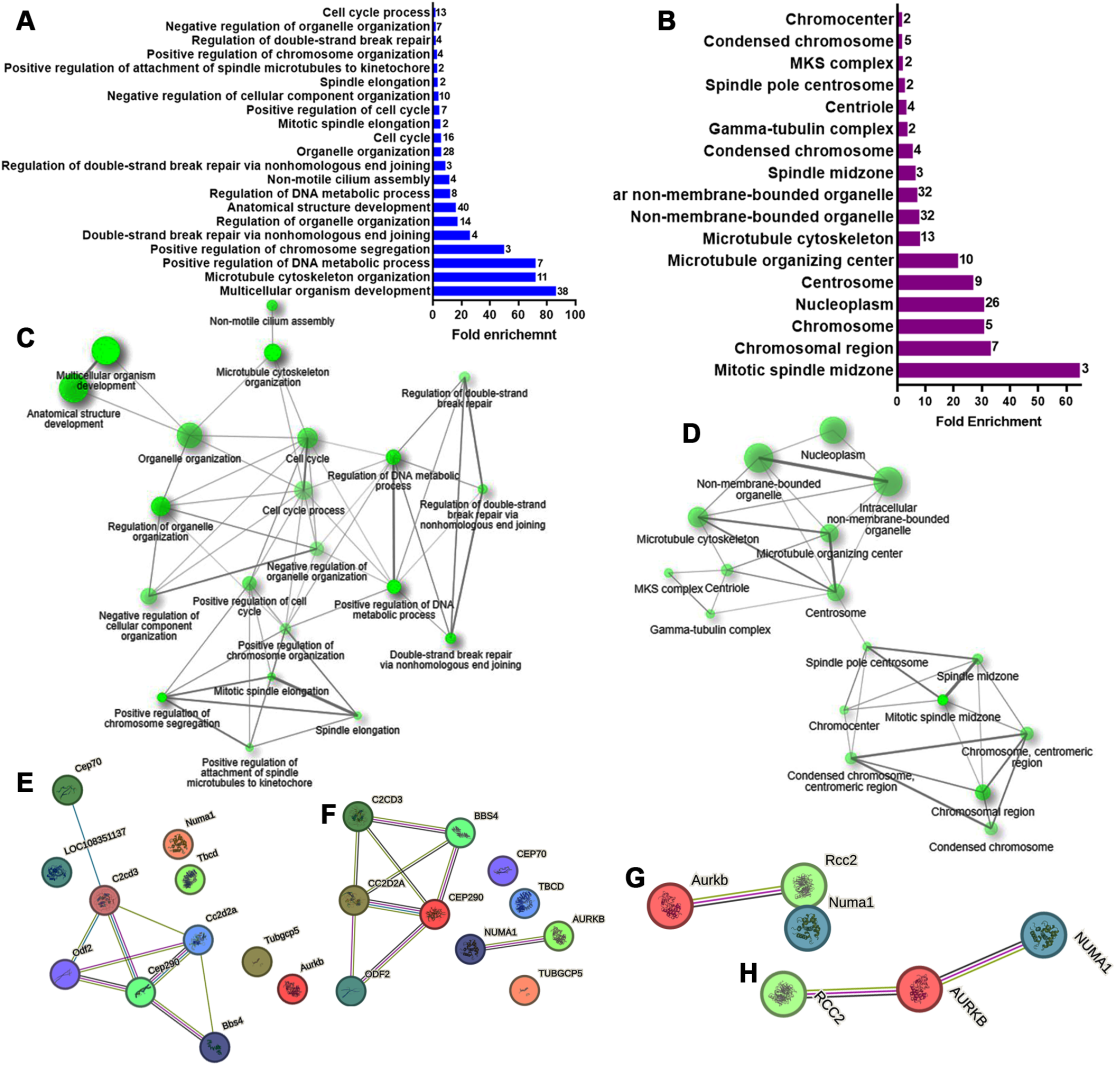
Gene ontology (GO) Biological Processes and Cellular Component enrichment analysis of up-regulated genes in E12 dorsal prosencephalon from diabetic dams. **(A, B)** Fold-enrichment graph showing 21 significantly enriched GO biological process terms (blue bars) and 17 cellular components terms (violet bars) in E12 tissue. Number within bars indicate the number of upregulated genes involved in each GO term (cut-off FDR P < 0.05). **(C, D)** Network clustering of GO biological process and cellular components terms. Nodes represent pathways; two nodes are connected if they share 20% or more genes. Darker nodes indicate greater statistical significance, larger nodes represent larger gene sets, and thicker edges indicate a higher gene overlap (overlap coefficient of at least 0.3). **(E, F)** Protein-protein interaction network for the GO term “Microtubule Cytoskeleton Organization” in rat and human, respectively. **(G, H)** Protein-protein interaction network for the GO term “Mitotic spindle midzone” in rat and human, respectively. Circles represent proteins. Interaction types are indicated by colored lines: green (curated databases), pink (experimentally determined), olive green (text mining), and black (co-expression).

Differentially expression analysis identified 247 genes (out of 24170) with significant changes: 111 were up-regulated and 136 down-regulated (**Figure 1D**; **Supplementary Tables 1, 2**). GO analysis of the biological process for the up-regulated genes revealed 21 enriched terms, primarily related to cell cycle and division. The most enriched terms included multicellular organism development (GO:0007275), microtubule cytoskeleton organization (GO:0000226), and positive regulation of DNA metabolic process (GO:0051054; **Figure 2A**, **Supplementary Table 3**). A network analysis showed that these terms were interconnected (**Figure 2C**). Notably, 57% and 85% of the enriched biological processes included Numa1 and Aurkb, respectively.

To refine the analysis, a STRING functional protein association network was generated for the second-most enriched process. In rats, this network revealed a node comprising six of the eleven proteins, excluding Numa1 and Aurkb (**Figure 2E**). However, in humans, a node containing only NUMA1 and AURKB was evident (**Figure 2F**).

GO analysis for cellular components identified 17 enriched terms, with the top three being mitotic spindle midzone (GO:1990023), chromosomal region (GO:0098687), and chromosome centromeric region (GO:0000775; **Figure 2B**, **Supplementary Table 4**). Numa1 and Aurkb were present in 53% and 82% of these terms, respectively. The resultant network demonstrated that the centrosome and spindle pole centrosome acted as key hubs. Interestingly, Numa1 and Aurkb were the only genes associated with the spindle pole centrosome (**Figure 2D**, **Supplementary Table 4**).

In rats, STRING analysis for mitotic spindle midzone term (GO:1990023) excluded Numa1 (**Figure 2G**). However, in humans, a unified network included the three up-regulated genes listed associated with this term (**Figure 2H**).

**Figure 3:**
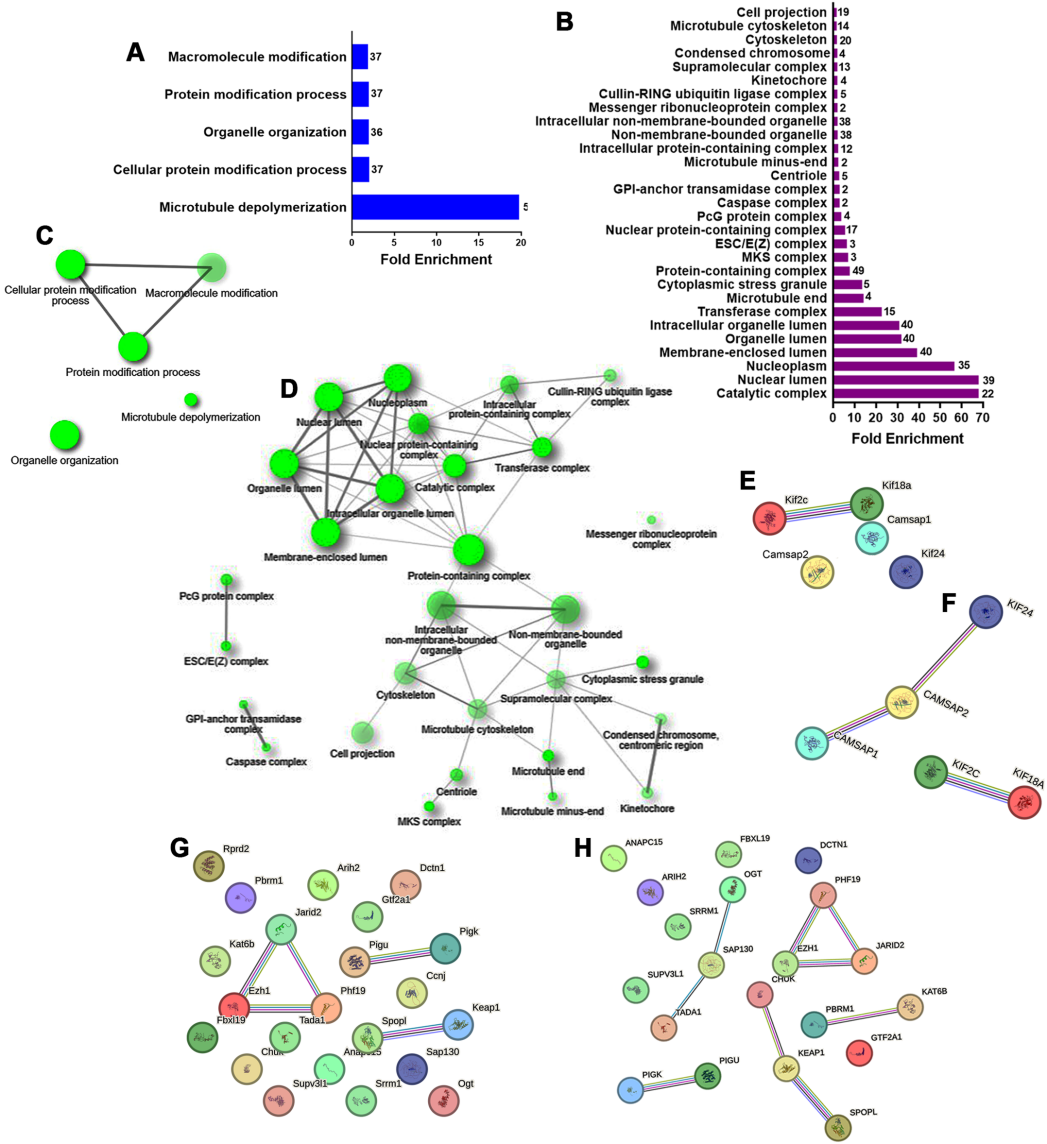
GO biological processes and cellular component enrichment analysis of the down-regulated genes in E12 dorsal prosencephalon from diabetic dams. **(A, B)** Fold-enrichment graph showing significantly enriched GO biological process (blue bars) and cellular components (violet bars) terms. Numbers within bars indicates the number of down-regulated genes involved in each term (cut-off FDR P < 0.05). **(C, D)** Network clustering of GO biological process and cellular component terms. Nodes represent pathways; two nodes are connected if they share 20% of genes. Darker nodes indicate greater statistical significance, larger nodes represent larger gene sets, and thicker edges indicate greater gene overlap (overlap coefficient of at least 0.3). **(E, F)** Protein-protein interaction network for down-regulated genes involved in the GO term “Microtubule depolymerization” in rat and human, respectively. **(G, H)** Protein-protein interaction network for down-regulated genes involved in the GO term “Catalytic complex” in rat and human, respectively. Circles represent proteins. Interaction types are indicated by colored lines: pink (experimentally determined), olive green (text mining), cyan (curated database), violet (protein homology), and black (co-expression).

For the down-regulated genes, GO analysis of biological process identified five affected terms: microtubule depolymerization (GO:0007019), cellular protein modification (GO:0006464), organelle organization (GO:0006996), protein modification (GO:0036211), and macromolecule modification (GO:0043412; **Figure 3A**). These terms included genes such as Kif18a, Kif2c, Kif24, Camsap1, and Camsap2, which are important for chromosome movement and cilia formation during mitosis (**Supplementary Table 5**). Network analysis showed that microtubule depolymerization and organelle organization were isolated terms (**Figure 3C**). STRING analysis in rats revealed a node with Kif2c and Kif18a, proteins with microtubule plus-end depolymerizing activity in mitotic cells (**Figure 3E**). In humans, an additional node included interactions between Kif24, Camsap1, and Camsap2, which are crucial for anchoring centrosomes and restricting cilia nucleation at centrioles (**Figure 3F**).

GO analysis of cellular components for down-regulated genes revealed 29 enriched terms (**Figure 3B**, **Supplementary Table 6**). The top three were catalytic complex (GO:1902494), nuclear lumen (GO:0031981), and nucleoplasm (GO:0005654). The resultant network linked 24 terms with the protein-containing complex in the center of the network (**Figure 3D**). STRING analysis revealed three nodes shared between rats (**Figure 3G**) and humans (**Figure 3H**). These nodes included: GPI-anchor transamidase complex (GO:0042765) with Pigu and Pigk; ESC/E(Z) complex (GO:0035098) with Ezh1, Phf19, and Jarid2; and cullin-RING ubiquitin ligase complex (GO:0031461) with Spopl and Keap1, and in humans CHUK.

### 3.2 Numa1 and Aurkb expression and spindle morphology

**Figure 4:**
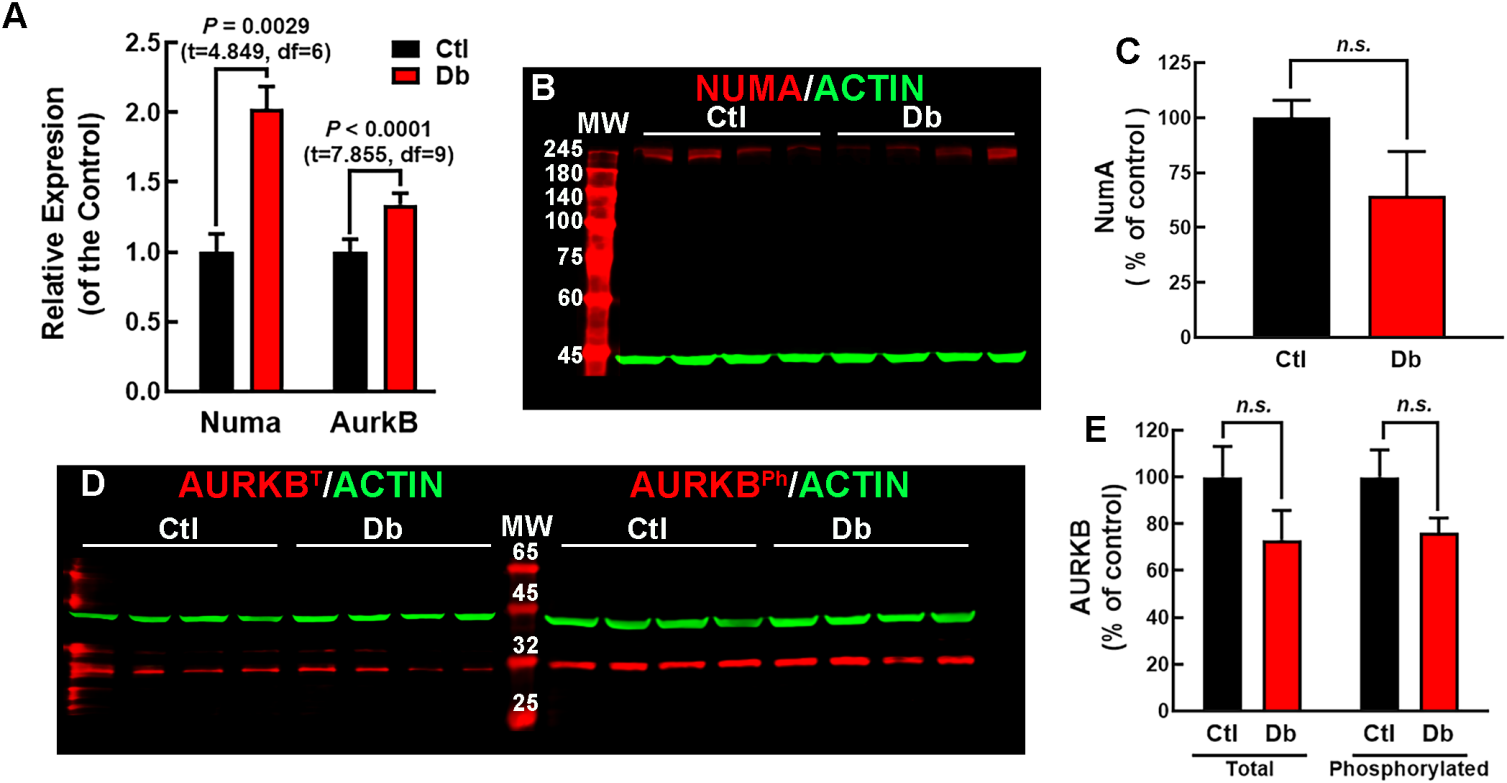
Expression analysis of NUMA and AURKB in E12 dorsal prosencephalon from control and diabetic rats. **(A)** Relative expression levels of Numa and Aurkb in control (Ctl, black bars) and diabetic (Db, red bars) groups, determined by qRT-PCR (2-ΔΔCT method). Data are shown as means ± S.E.M. (N = 4). P values were obtained using the Student´s t-test. **(B)** Western blot analysis of NUMA (~238 kDa, red bands) and actin (~42 kDa, green bands) in Ctl and Db groups samples (N = 4). MW = molecular weight marker. **(C)** Quantitative analysis of NUMA/ACTIN fluorescence intensity expressed as a percentage of the Ctl. No significant difference (n.s.) was detected (t- test). **(D)** Western blot analysis of total (AURKBT) and phosphorylated AURKB (AURKBPh ~35 kDa, red bands) with actin (~42 kDa, green bands) in Ctl and Db groups (N = 4). MW = molecular weight marker. **(E)** Quantitative analysis of total and phosphorylated AURKB/ACTIN fluorescence intensity. No significant difference (n.s.) was detected (test).

The bioinformatics and the reported increased neurogenic markers in embryos from diabetic rats and mice at E14 and E11.5, respectively (11–13), suggest impaired cell division. Two key genes involved in the planar division, Numa1 and Aurkb, were identified as up-regulated. Since these two genes were involved in more than 50% of the enriched terms significantly affected, emerging as key regulators of mitotic spindle architecture and chromosome segregation. Furthermore, they have been shown that defects in spindle orientation and asymmetric cell division lead to abnormal neurogenesis, which is a hallmark of neurodevelopmental impairments in diabetic conditions (26, 28). qRT-PCR validated their increased expression, showing a 2-fold and a 1.3-fold increase for Numa1 and Aurkb, respectively, in the Db vs the Ctl group (**Figure 4A**). However, protein levels and AURBT232ph did not showed significant changes (**Figures 4B–E**).

**Figure 5:**
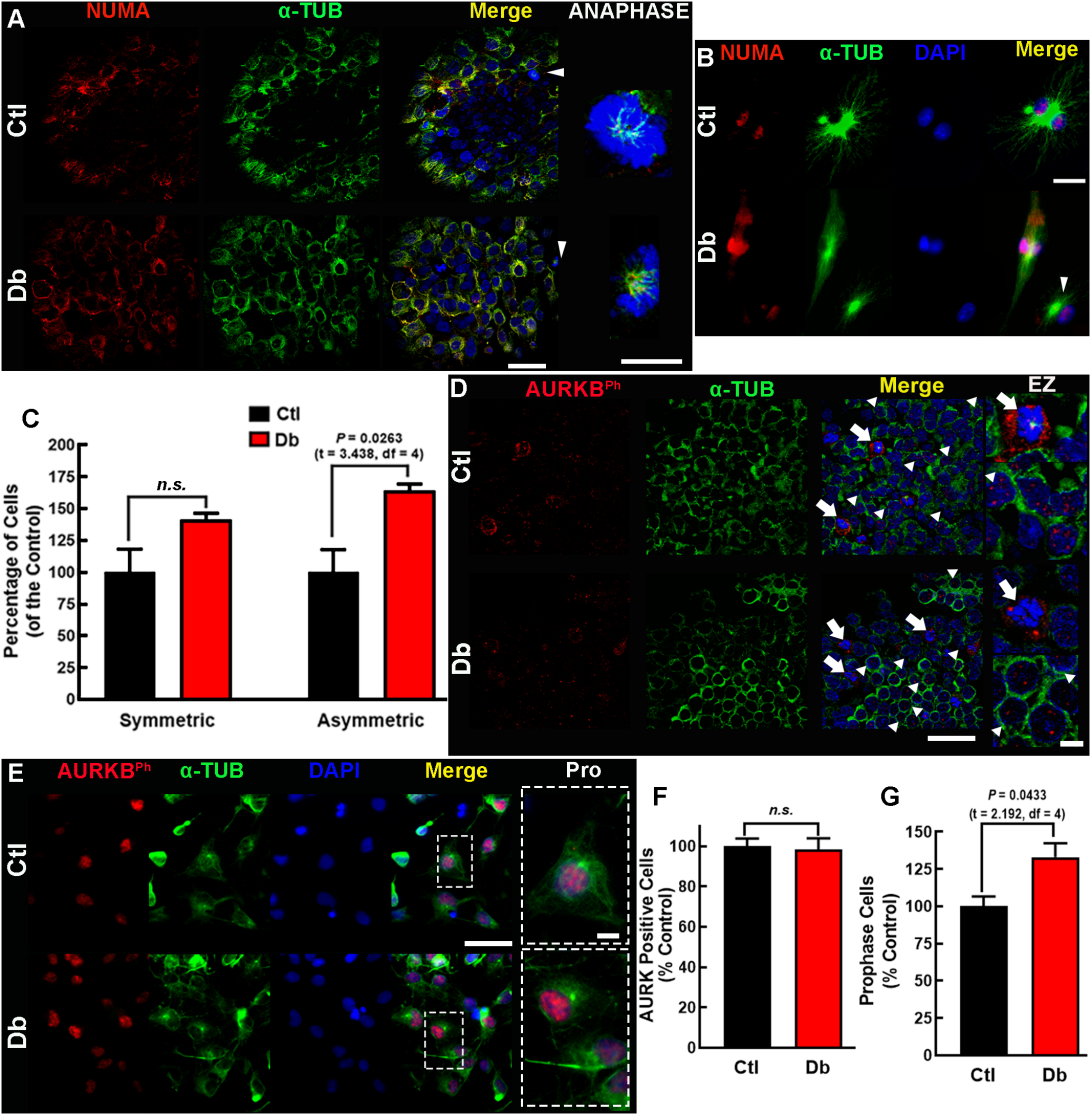
In-face view and *in vitro* analysis of NUMA and AURKB expression and distribution. **(A, B)** Representative micrographs (60×) of NUMA (red) and α-tubulin (α-TUB, green) in the ventral zone of E12 dorsal prosencephalon tissue **(A)** and cultured neural stem cell **(B)** from control (Ctl) and diabetic (Db) groups shown in single and merged channels. On the right, zoomed (200×) images of cells in anaphase (white arrowheads). Nuclei are stained with DAPI (blue). **(C)** Quantitative analysis of symmetric and asymmetric NSC divisions, expressed as percentage of Ctl ± SEM (n = 4). P value obtained using the t-Student test. n.s., no significance. **(D, E)** Representative micrographs (40×) of phosphorylated AURKB (AURKBph, red) and α-tubulin (α-TUB, green) immunostaining in E12 dorsal prosencephalon **(D)** and cultured NSC **(E)** presented in single and merged channels. On the right, zooms (EZ; 200×) for cells in anaphase (white arrowheads) and late telophase (arrows). Nuclei stained with DAPI are shown in blue. **(F)** Quantitative analysis of total and **(G)** prophase AURKBPh-positive NSC, expressed as percentage of Ctl ± SEM (n = 4). P value obtained using the Student´s t-test. n.s., no significance. Scale bar = 25 μm and for zoomed images 10 μm.

Immunofluorescence analysis of en-face mounted explants revealed a belt-like distribution of NUMA1 in most cells of both groups, indicative of prometaphase. However, the Db group exhibited a higher number of cells in prometaphase, suggesting an increased entry in mitosis (**Figure 5A**). In metaphase/anaphase cells, α-TUB staining showed significant spindle abnormalities in the Db group, including monopolar spindles, compared to the typical astral spindles in the Ctl group (**Figure 5A**).

Similar findings were observed in cultured E12 NSC, where monopolar spindles were prevalent in the Db group (**Figure 5B**). Analysis of NUMA1 distribution indicated a significant increase in asymmetric cell division in the Db group (1.63-fold vs Ctl), with no significant changes in symmetric divisions (**Figure 5C**). These results suggest aberrant spindle morphology and premature asymmetric cell division in the cortical neuroepithelium of embryos from diabetic dams.

In prometaphase, AURKBT232ph is typically localized at mid-zone chromosomes, stabilizing spindle elongation and cleavage furrow formation (43). In the Ctl group explants, AURKBT232ph displayed a distinct pattern of four nuclear dots and peripheral staining around chromosomes, with corresponding α-TUB staining at the center. In the Db group, this pattern was disrupted, with irregular dots and reduced peripheral staining of AURKBT232ph accompanied by absent α-TUB staining (**Figure 5D**). Cultured NSC showed no changes in the number of AURKBT232ph-positive cells, but a significant increase in cells undergoing prophase was observed (**Figures 5E–G**).

**Figure 6:**
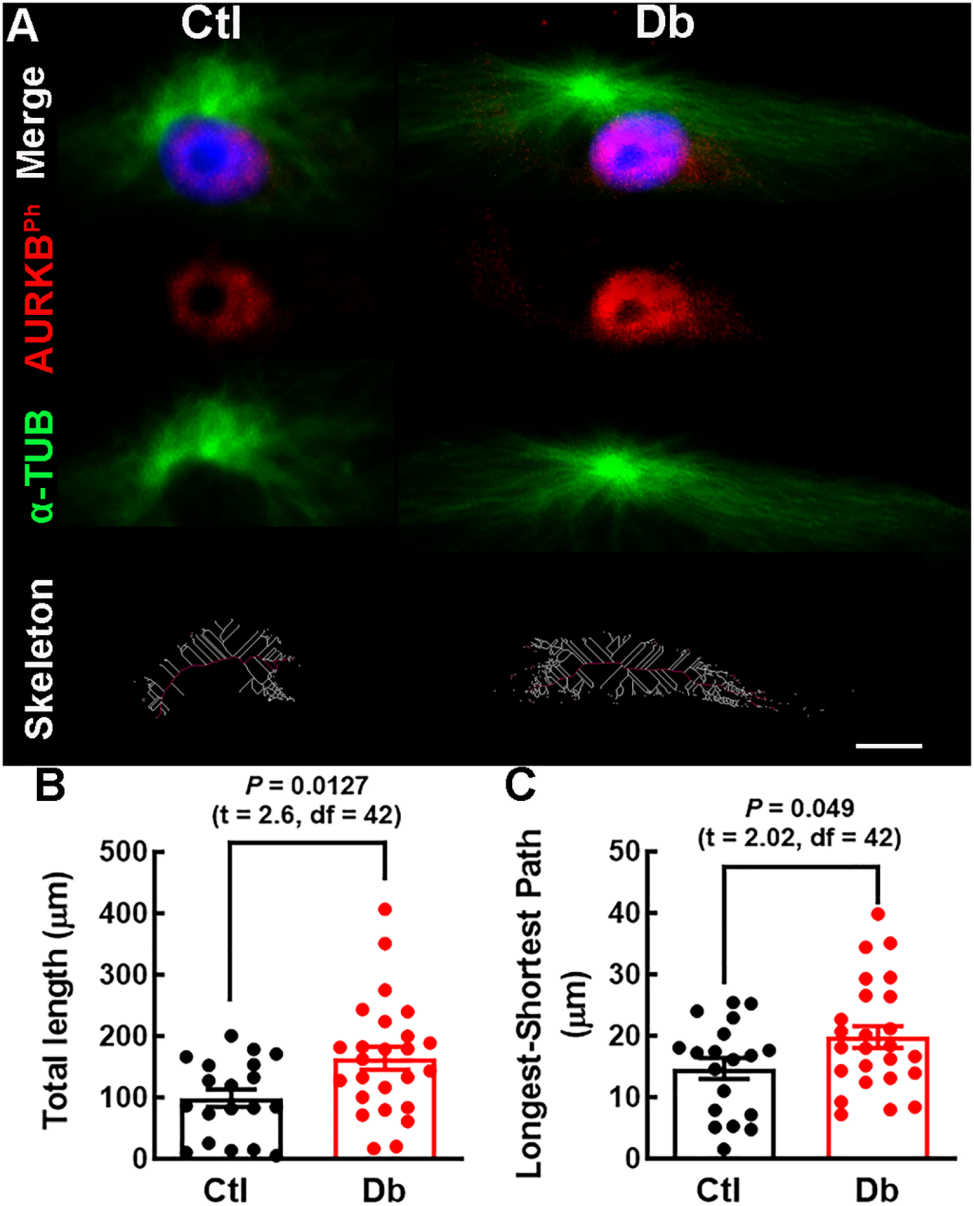
Mitotic spindle structure analysis in cultured E12 neural stem cells. **(A)** Representative micrographs (40×) of α-TUBULIN (α-TUB, green) staining of mitotic spindles in cultured E12 NSC. Nuclei are stained with DAPI (blue). Bottom panel; spindle skeleton (white lines) and the longest-shortest path (red line) representing spindle length in late prophase NSC. **(B, C)** Quantitative analysis of total spindle length (White lines) and mitotic spindle size (red line) in control (Ctl, black bars and dots) and diabetic (Db, red bars and dots) groups. Data points represent individual cells from three experiments. P values were obtained using the Student´s t-test. Scale bar = 2 μm.

Given the enrichment of microtubule depolymerization process in down-regulated genes, we analyze mitotic spindle length using α-TUB staining. The Db group exhibited longer and more robust spindles (**Figures 6A–C**), supporting the bioinformatic prediction of impaired microtubule depolymerization.

## 4 Discussion

This study aimed to understand the molecular disruptions associated with maternal diabetes and their potential impact on early corticogenesis, specifically focusing on cell cycle regulation, spindle dynamics, and neurogenesis.

The bioinformatic analysis of the RNA-seq data from embryonic samples without neural tube defects at E12 provides significant insights into the impact of maternal diabetes on gene expression in the developing dorsal prosencephalon.

Although mating was controlled as carefully as possible, the heterogeneity observed may stem from slight differences in the embryonic stage between litters and stochastic fluctuations in gene expression during early corticogenesis (44, 45). Such variability is expected in developmental studies. Despite sample variability, as highlighted by the PCA and hierarchical clustering analyses, differential gene expression analysis identified 111 up-regulated and 136 down-regulated genes out of 24,170. These findings consistently point to disruptions in cell cycle regulation and spindle dynamics as key consequences of maternal diabetes.

The GO analysis of the up-regulated genes revealed their involvement in key biological processes and cellular components related to cell cycle regulation and mitosis. Enriched terms such as multicellular organism development, microtubule cytoskeleton organization, and positive regulation of DNA metabolic processes suggest that these genes are critical in maintaining proper cell division and genome stability during neurodevelopment. The genes Numa1 and Aurkb emerged as central regulators within these processes. STRING analysis highlighted their inclusion in conserved protein interaction networks in rats and humans, indicating that the underlying molecular mechanisms of cell cycle regulation may be translatable between species.

In contrast, the down-regulation of genes such as Kif18a, Kif2c, and Kif24 are key players in microtubule dynamics, and a decreased expression could disrupt the delicate balance of symmetric and asymmetric cell division necessary for proper neurogenesis and, consequently, neuroblast migration, ultimately affecting corticogenesis. Interestingly, evidence of impaired migration has been previously reported in offspring from diabetic dams (10). The GO-cellular component analysis further supports the hypothesis that maternal diabetes disrupts critical aspects of the mitotic cell cycle, particularly those associated with spindle assembly and chromosome segregation. Disruption in networks such as catalytic complexes, nuclear lumen, and membrane-enclosed lumen components may affect intracellular signaling and gene expression regulation, contributing to neurodevelopmental deficits in offspring.

The identification of similar protein interaction networks in rats and humans involving proteins like KIF2C, KIF18A, PHF19, JARD2, and EZH1 underscores the potential relevance of these findings to human development. Highlighting that the molecular machinery might be affected in both species due to maternal diabetes, supporting the relevance of the results obtained and highlighting the importance of evaluating early corticogenesis in animal models of maternal diabetes.

Microtubules are essential for mitotic spindle architecture, which evolves as neurodevelopment progresses. In early neurogenesis (E12 in rats), astral spindle morphology is typical and associated with symmetric cell division. By the neurogenic peak (E14 in rats), cell division predominantly switches to an asymmetric mode characterized by inner spindle morphology (24). This transition from symmetric to asymmetric division is critical for defining brain cytoarchitecture and size (21, 46, 47).

Our experimental findings show that maternal diabetes affects mitotic spindle morphology and cell division dynamics in the E12 cortical neuroepithelium. At this stage, the dorsal prosencephalon is primarily populated by NSC, and the shift from symmetric to asymmetric division initiates deep-layer neurogenesis (46). The altered expression of Numa1 and Aurkb in the Db group could likely promote an early shift toward asymmetric cell divisions. This premature switch can be associated with increased neurogenic markers previously reported in rodent embryos from diabetic mothers at the neurogenic peak (8, 9). The early commitment of NSCs to neural differentiation could explain the depletion of the NSC pool previously reported (7, 8), which is necessary for later stages of brain development and could also explain the increase in asymmetric cell proliferation observed in this study.

Although Numa1 and Aurkb expression levels were increased in the Db group, this did not correspond to higher protein levels or phosphorylation of AURKBT232, suggesting possible post-transcriptional regulation. However, immunofluorescence analysis supported the presence of mitotic defects. The belt-like distribution of NUMA1 exhibited in Db explants, characteristic of prometaphase cells, indicates potential mitotic delay or dysregulation. Such delays have been linked to abnormal spindle architecture, evidenced by an increased prevalence of monopolar spindles in Db explants and cultured NSCs observed here, contrasting with the typical astral spindle in controls (29, 48).

The spindle assembly checkpoint may be compromised in the Db group, leading to delays in chromosome segregation as cells attempt to correct spindle attachment errors (49). This opens avenues for exploring the interplay between NSC gene expression, protein function, and cell-cycle regulation in maternal diabetes and neurodevelopment.

The observed changes in mitotic spindle morphology may result from impaired microtubule depolymerization, supported by the bioinformatic enrichment of related terms among the down-regulated genes. This aspect must be further studied since it is known that defects in microtubule depolymerization compromise spindle integrity and orientation, which are critical for symmetric and asymmetric cell divisions (24).

Disrupted AURKBT232ph in Db explants suggests potential chromosome segregation issues and cleavage furrow formation (50, 51). In controls, AURKBT232ph displayed a punctuated organized pattern aligned with the chromosomal midzone, facilitating spindle elongation and chromosome separation. This pattern was disrupted in Db samples, indicating potential spindle instability and misalignment, which could interfere with chromosome segregation and cytokinesis. Interestingly, changes in AURKBT232ph have been associated with defective spindle positioning and misorientation during cell division, resulting in premature neurogenesis and differentiation due to early asymmetric cell division processes (49–51).

The chromosomal passenger complex, comprising AURBK, the inner centromere protein, borealin, and survivin, stabilizes microtubules and regulates the spindle assembly checkpoints (52–55). Disrupted AURKBT232ph patterns in the Db embryos may reflect attempts to correct microtubule attachment errors as a mechanism in embryos without neural tube defects to ensure complete development and survival (49, 56). AURKB is considered the central regulator of the error correction process of kinetochore-microtubule attachments through the phospho-regulation of KIF2C, a kinesin-13 family member involved in microtubule depolymerization (57–59), a gene downregulated in the Db group. Thus, in a microtubule low-tension situation, as suggested by longer spindles observed in this study, AURKB may promote microtubule destabilization, promoting error correction. However, further studies are needed to elucidate its precise role during the corticogenesis of embryos exposed to high glucose.

Quantifying mitotic spindle length revealed longer and more robust spindles in the Db group, consistent with impaired microtubule depolymerization. Insufficient depolymerization can lead to aberrant spindle geometry, affecting cell fate decisions and reducing the progenitor pool necessary for cortical expansion (29, 49, 60). The shift toward asymmetric divisions may contribute to long-term neurodevelopmental deficits previously reported in children from diabetic mothers.

The mitotic abnormalities observed in our study are particularly significant because NSCs in the VZ undergo tightly regulated symmetric and asymmetric divisions to maintain balanced neurogenesis and gliogenesis. Normally, symmetric divisions at early neurodevelopmental stages expand the NSC pool, while asymmetric divisions promote time-dependent differentiation into neurons and glia (13). Our findings indicate that maternal diabetes drives this balance toward asymmetric division earlier than expected, which could lead to premature differentiation and an insufficient progenitor pool for later cortical development (21).

This premature neurogenic commitment has been observed in previous studies on diabetic embryopathy. For instance, increased neurogenic markers and reduced neural progenitor proliferation were reported in E14 embryos from diabetic rats (7, 9), suggesting an early exhaustion of progenitor cells. Interestingly, altered mitotic spindles, as observed in our study, are known to affect interkinetic nuclear migration, which is crucial for NSC self-renewal (20). Disruptions in this process may lead to aberrant neuronal positioning and could contribute to the cortical thinning and cytoarchitectural defects reported in the offspring of diabetic mothers (10). Furthermore, the depletion of the NSC pool and premature neurogenesis could have profound consequences for cortical function and behavior. Indeed, reduced cortical progenitor proliferation in diabetic pregnancies has been linked to altered cortical thickness, function, and impaired neuronal connectivity (10, 28). These structural deficits may underlie the cognitive and motor impairments observed in children of diabetic mothers, such as deficiencies in working memory, attention, and executive function (61).

The precise regulation of mitotic spindle orientation and cell cycle progression in NSCs is crucial for maintaining the balance between self-renewal and differentiation. Disruptions in mitotic spindle orientation and cell cycle progression have been linked to neurodevelopmental disorders such as microcephaly and autism spectrum disorders (ASD) (62, 63). Furthermore, aberrant mitotic spindle orientation has been implicated in cortical thinning, misplacement of neurons, and disrupted neuronal connectivity (62). Given that Aurkb and Numa1 play crucial roles in spindle assembly and chromosome segregation (29), their dysregulation under diabetic conditions may contribute to similar neurodevelopmental deficits. Our data suggest that maternal diabetes disrupts self-renewal and differentiation by promoting early asymmetric divisions, possibly depleting the NSC population prematurely, as observed in E14 telencephalons (7). This conclusion is supported by increased expression of Aurkb and Numa1, genes involved in mitotic spindle organization and chromosome segregation, alongside structural abnormalities in spindle morphology.

One mechanism potentially linking maternal diabetes to mitotic dysregulation is oxidative stress, which can impair NSC proliferation and differentiation (27). Hyperglycemia-induced oxidative damage may alter spindle microtubule stability and chromosome segregation, thereby increasing mitotic errors. Additionally, epigenetic modifications such as changes in DNA methylation patterns have been reported in the brains of offspring from diabetic pregnancies, which may influence gene expression programs involved in neurogenesis (26, 64, 65).

Our study has certain limitations, including the use of only one developmental window. However, previous studies by our group and others indicate that, under this same model, findings related to developmental timing and postnatal outcomes collectively offer insights into both prenatal and postnatal cortical development. Moreover, although our transcriptomic and immunofluorescence analyses provide strong evidence of altered mitotic regulation, additional functional assays, such as time-lapse imaging of neural progenitor divisions or *in vivo* lineage tracing, would strengthen our conclusions.

Future studies should explore whether pharmacological or molecular interventions aimed at stabilizing the mitotic spindle or mitigating oxidative stress can offset these effects. Single-cell RNA sequencing could also provide deeper insights into the fate of prematurely differentiated neural progenitors. Moreover, research on microtubule dynamics during mitosis is warranted, given that genes such as Kif18a, Camsap2, Kif24, Camsap1, and Kif2c, which were found to be down-regulated, are involved in microtubule depolymerization together with Numa (65). Employing *in vivo* conditional knockout models or *ex vivo* shRNA approaches would be valuable for dissecting the mechanistic role of these genes in NSC function and corticogenesis.

Clinically, our findings underscore the importance of maternal glycemic control during pregnancy to prevent early neurodevelopmental abnormalities that may predispose offspring to cognitive and behavioral disorders. Understanding the molecular basis of maternal diabetes-induced neurodevelopmental impairments could inform therapeutic strategies. Given that metabolic disorders such as gestational diabetes are increasing in prevalence, identifying early biomarkers of neural dysfunction may aid in developing preventative interventions aimed at improving neurodevelopmental outcomes in affected offspring.

In conclusion, maternal diabetes disrupts the expression of genes critical for microtubule dynamics, spindle formation, and cell division symmetry, thereby impairing early corticogenesis. Aberrant mitotic spindle formation and premature asymmetric cell division may underlie the neurodevelopmental vulnerabilities observed in the offspring of diabetic mothers. The similarities in protein interaction networks between rats and humans highlight the importance of using *in vivo* models to advance our understanding of these mechanisms and their implications for human health. Further research into the molecular pathways affected by maternal diabetes may identify potential therapeutic targets to prevent neurodevelopmental impairments associated with gestational diabetes.

## Data Availability

The original contributions presented in the study are included in the article/**Supplementary Material**, further inquiries can be directed to the corresponding author/s.
